# Anti-Coccidial Effect of Rumex Nervosus Leaf Powder on Broiler Chickens Infected with Eimeria Tenella Oocyst

**DOI:** 10.3390/ani11010167

**Published:** 2021-01-12

**Authors:** Mohammed M. Qaid, Saud I. Al-Mufarrej, Mahmoud M. Azzam, Maged A. Al-Garadi, Hani H. Albaadani, Ibrahim A. Alhidary, Riyadh S. Aljumaah

**Affiliations:** 1 Animal Production Department, College of Food and Agriculture Sciences, King Saud University, Riyadh 11451, Saudi Arabia; salmufarrej@ksu.edu.sa (S.I.A.-M.); mazzam@ksu.edu.sa (M.M.A.); malgaradi@ksu.edu.sa (M.A.A.-G.); hsaeed@ksu.edu.sa (H.H.A.); ialhidary@ksu.edu.sa (I.A.A.); rjumaah@ksu.edu.sa (R.S.A.); 2 Veterinary Department, Faculty of Agriculture and Veterinary Medicine, Thamar University, Dhamar 13020, Yemen

**Keywords:** anti-coccidial effect, broiler chicken, *Eimeria tenella*, *Rumex nervosus* leaf powder

## Abstract

**Simple Summary:**

*Eimeria tenella* pathogens belong to the Eimeriidae family and the Apicomplexa phylum, which invades the cecal epithelium of birds, resulting in massive injury and economic loss. We evaluated the ameliorative effect of *Rumex nervosus* (RN) leaf powder against *E. tenella*-induced coccidiosis in broiler chickens. Chickens infected with *E. tenella* were treated with 1, 3, and 5 g/kg RN, respectively. Salinomycin sodium (Sacox^®^), an anti-coccidial agent, was used as a reference drug. Results have shown that RN contains Gallic acid and 13 phytochemicals, which require further investigation in vitro or in vivo to ascertain whether the anti-coccidial activity, if there, is a direct or indirect link to reduce the number of fecal oocysts in the bird. The lesion score and bloody diarrhea were also decreased after infection. Moreover, the coccidial challenge adversely affected (*p* < 0.05) the performance measurements in the RN- and Sacox-treated groups compared with the uninfected–unmedicated control (NC) group. Interestingly, these parameters were positively affected by natural and synthetic treatments compared with infected–unmedicated control (PC); however, the values were not significant. In conclusion, RN at the highest dose is a promising shrub with a moderate anti-coccidial activity when used to cure avian coccidiosis.

**Abstract:**

Coccidiosis a huge economic burden in poultry farms where the pathogen *Eimeria* harms animal well-being and survival. Besides synthetic anti-coccidial drugs, natural herbs appear to be an alternative way to prevent avian coccidiosis. *Rumex nervosus* (RN), a phytogenic shrub, has received considerable attention in recent years due to its significant anti-microbial effects; however, limited knowledge exists about its potential anti-coccidial functions. This study was conducted to evaluate the prophylactic and therapeutic activities of RN leaf powder in broilers infected with *Eimeria tenella*. Infected chickens received a commercial diet containing 1, 3, or 5 g RN powder/kg diet compared to infected broilers that treated with Sacox (PC) or compared to uninfected broilers that received a commercial diet alone (NC). Results showed that RN powder significantly (*p* < 0.05) reduced the lesion scores and suppressed the output of oocysts per gram (OPG) in chickens’ feces. Although RN was unable to minimize the weight gain loss due to emeriosis, RN at level 1 g improved the feed conversion ratio. Therefore, RN powder, at 5 g, possesses moderate anti-coccidial effects and hence could be used to treat avian coccidiosis in field conditions; however, further studies are required to investigate, in vitro or in vivo, the anti-coccidial potential of active ingredients.

## 1. Introduction

Coccidiosis is an intracellular protozoan disease that affects the hygiene and performance of the majority of animals and birds. Coccidian protozoa belonging to several genera and species cause coccidiosis. The genus *Eimeria* affects birds, cattle, rabbits, and other mammals, and the genus *Isospora* is known to infect dogs and cats [1]. The protozoan Eimeria that belongs to the phylum Apicomplexa causes avian coccidiosis. Avian coccidiosis is one of the most serious poultry diseases and affects the worldwide poultry industry. More than 50 billion poultry are raised as meat source each year, accounting for more than a third of protein foods for humans [2]. However, coccidiosis causes large economic losses of up to USD 3 billion dollars worldwide [1,3]. Poor performance and high mortality and morbidity of chickens, associated with medication expenditure, subsequently contribute to the financial burden. The overall prevalence of coccidiosis in Riyadh, Saudi Arabia, in a year was reported at 75% among domestic rabbits [4] and 80% among house-reared chicks [5]. Ref. [6] reported that younger chicks were more susceptible than older chicks to emeriosis. *Eimeria acervulina*, *E. maxima*, *E. tenella*, and *E. praecox* are often found in broiler chickens.

Dietary supplementation of *Curcuma longa* can be used to enhance coccidiosis resistance through increased body weight gain (BWG), decreased lesion score of the gut, and reduced shedding of fecal oocysts [7]. Moreover, a natural product, betaine, can reduce the effects of coccidian challenge [8]. Berberine has been reported to exert a protective effect on chicks’ cecum infected with *E. tenella* [9]. Mixtures of essential oil (eugenol, thymol, and piperine) and vitamin D_3_ may be used as an anti-coccidial feed supplement in coccidian-infected broilers [10]. This supplement has a positive effect on the BWG and feed conversion ratio (FCR) of birds due to improved digestibility of nutrients by stimulating digestive enzymes, increasing beneficial microflora, and reducing intestinal lesions caused by coccidial protozoa. Various chemical anti-coccidial drugs such as Amprolium, Monensin, Diclazuril, and Sacox are widely used in the poultry industry and have effective control of avian coccidiosis [11]. Salinomycin is a standard coccidiostat monovalent ionophoric therapeutic drug that disrupts alkali ions gradients across the cell membrane of Eimeriosis across interfering with potassium potential transmembrane and promoting K+ ions efflux from cytoplasm and mitochondria. As a result, Salinomycin acts at the early stages of the parasite life cycle and is most useful as a preventative than a therapeutic drug. Here, Salinomycin used with the diet as preventative or coccidiostat rather than as coccidiocidal drug. The coccidiocidal such as Toltrazuril (Baycox^®^) as one of the triazines used during outbreaks due to Toltrazuril acts on intracellular stages of the life cycle that are undergoing schizogony and gamogony leading to control parasite in broilers with just a single 2-day treatment course [12,13,14]. However, the incidence of drug resistance from parasites and the consumers prefer residue-free meats have prompted a number of researchers to suggest and develop alternative agents [15,16].

In recent years, some phytogenic feed additives have been considered to be effective in protecting chickens from coccidia-induced injury. [17] reviewed the interest in medicinal plants and their derivatives as coccidiostat replacements for use in poultry production. Numerous herbs and their extracts, including artemisinin (*Artemisia* species) [18], betaine (*Beta vulgaris)* [19], Echinacea species (American coneflower, purple coneflower) [20], *Aloe vera* [21], *Azadirachta indica* (Neem) [22], *Emblica officinalis* [22], and mushrooms [23], have been investigated for their anti-coccidial properties. These herbs produce bioactive phytochemical compounds that act against induced eimeriosis and are harmless to poultry meat consumers.

Further studies reveal that researchers are exploring a low-cost natural product with reduced side effects against emerioisis [24,25]. Considering that herbs and other biological constituents are safe, nutritious, and therapeutic, the use of natural anti-oxidants has recently received considerable attention [26,27,28].

Ithrib *(R. nervosus* Vahl) is a perennial herbaceous plant, available year round, and native extensively to some Eastern African countries and the Arabian Peninsula. It is distributed in Yemen, Saudi Arabia, Somalia, Ethiopia, Tanzania, and Kenya [29,30]. Consequently, those countries need alternative anti-coccidial drugs that able to control the avian coccidiosis disease without affecting the production efficiency or human health through either poultry company customers or end-user consumers [25,26,29].

It is an ethnomedicinal plant originally belonging to the Polygonaceae family. The root, stem, leaf, and metabolites of *Rumex nervosus* (RN) contain essential phytochemical ingredients such as carbohydrates, vitamins, sterols, glycosides, triterpenes, saponins, tannins, and flavonoids, which possess a variety of pharmacological and biological activities and potential applications in medicine [30,31,32,33]. Recent studies show that RN leaf extracts have anti-coccidial and anti-inflammatory activities [34]. RN has anti-oxidant properties and improves the performance of broiler chickens [35]. It is used as traditional therapy for several diseases and functions such as an anti-bacterial [29] and anti-parasitic [36] agent. There are pharmacognostical study of RN growing in Yemen reported by [30] reported that the ethanolic extract of RN considered as non-toxic up to a dose of 7.1 g/kg b.wt, and have cytotoxic activity with varying degrees of potency against human cancer cell line. In addition, [30] reviewed that RN have many traditionally uses and treated multifaceted cases in human such as many inflammatory diseases, wounds, diarrhea, typhus, rabies and skin disorders. As a result, RN as the natural medicinal herb has fewer adverse effects and a lower estimated cost, as opposed to synthetically produced chemical compounds [35]. Diarrhea and vomiting are side effects of RN in children. Thus, contraindication administered for children and the Red teff porridge considered antidotes [37]. The shrubs belonging to the Polygonaceae family may comprise a large amount of oxalic acid. Consuming a large amount of oxalic acid contributes to hyperoxaluria that leads to the calcium oxalate stone formation, and hypocalcemia due to reaction them with plasma calcium and precipitate insoluble calcium oxalate in liver, kidneys, heart, blood vessels, and lungs [38]. However, to date, there is limited or no evidence of RN leaf powder supplementation of diets used for coccidiosis therapy. Therefore, this study was conducted to evaluate the efficacy of different concentrations of RN leaf powder supplementation used as a dietary anti-coccidial and growth promoter agent in broiler chickens experimentally challenged with *E. tenella*.

## 2. Materials and Methods

### 2.1. Ethical Approval

Adequate measures were taken to minimize pain or discomfort of the birds, and the study procedures were approved by the Departmental Ethics, Methodology and Welfare Studies board, King Saud University (KSU), Kingdom of Saudi Arabia (Ethical committee approval No. KSU-SE-20-44).

### 2.2. Housing, Infection, and Experimental Design with Broiler Chickens

A total of 150 one-day-old hatched broiler chickens (Ross 308) were obtained from the local hatchery, AL-Khumasia Feed and Animal Products Company, Riyadh, Saudi Arabia. Birds were then randomized to six treatment groups using a completely randomized design. Each group consisted of 25 birds with five replicates per group and 5 birds per replicate. The starter (1 to 21 d) and finisher (22 to 28 d) diets were provided according to the Ross 308 guideline (Table 1). Birds were fed with commercial diets at 1, 3, and 5 g RN/kg diet in groups 1, 2, and 3, respectively. Group 4 birds were fed with basal diets plus 66 mg Sacox. The 66 mg Salinomycin/kg diet were used as standard coccidiosis drug supplemented with diet based on previous studies such as [12,13].

Group 5 was a positive control (PC), i.e., infected–unmedicated control group, in which birds were challenged with *E. tenella* oocysts without any addition of RN in their diet. Group 6 was the negative control (NC), i.e., an uninfected–unmedicated control group, in which birds have not exposed to *E. tenella* oocysts without any addition of RN to the diet. The overall study duration was 28 d, and the birds were reared in an environmentally controlled house in a cage measuring 58 cm length × 50 cm width × 35 cm height. The temperature and relative humidity were 33 °C and 65% up to 5 d of age and were reduced gradually to 24 °C and 50% up to 21 d of age. The “23-h-on and 1-h-off” photoperiod was used. Feed and water were provided on an ad libitum basis.

The *E. tenella* oocysts used in this study were kindly provided by collaborators from the Department of Zoology, College of Science, KSU, Riyadh, Saudi Arabia. Unsporulated oocysts were collected from the feces of chickens that were naturally infected on the 7th d after infection. These oocysts were sporulated, purified, and preserved in 2.5% potassium dichromate at 25 °C for 72 h and then stored at 4 °C until use [39]. Identification of the species of the sporulated oocysts was based on morphological characteristics and morphometry as described by [35], as well as the location of lesions. In addition, sporulated oocysts were passed twice over 2 weeks of age in healthy chickens before being used to activate the parasite and confirm the placement of the lesions. Oocysts were then collected, sporulated, washed, and adjusted to 1 × 10^4^/100 µL/bird. A total of 10,000 sporulated oocysts were administered orally (gavage) to each experimental chicken in 1 mL of distilled water [40].

### 2.3. RN Leaf Powder Preparation and Its Nutritional Composition Analysis

RN Vahl was collected from the valleys and mountains around the village of Bait Al-Aqra, Ibb region, Yemen. RN exsiccatae registration No. 23033, registered by botanist (herbarium specialist): Dr. Jacob Thomas at the Department of Botany, Faculty of Science at King Saud University. The harvested RN leaves had been air-dried every five hours for 15 days under sunlight. Then, the dried leaves were ground to a fine powder (particle size; 0.25–0.30 mm) using a blender in our laboratory. After that, grinding leaves were mixed with the diets of broilers at concentrations of 1, 3, and 5 g of RN/kg diet. Proximate analysis was conducted to determine the nutritional value of RN as described by [41]. High-performance liquid chromatography (HPLC) analysis was used for the identification, separation, and dosing of chemical compounds in the RN extract mixture. Gas chromatography-mass spectrometry (GC-MS) test was used for detecting the chemical composition as described by [42].

### 2.4. Anti-Coccidial Evaluation

To evaluate the anti-coccidial index (ACI) or efficacy of RN leaf powder, bloody diarrhea, fecal oocysts per gram, relative BWG, lesion scores, and survival rate were determined as anti-coccidial parameters based on the formula described by [43], and then the ACI value was determined as described by [44]. In brief, the ACI value below 120 was evaluated to be an inactive anti-coccidial effect, 120 to 140 as mild or slight, 140 to 160 as moderate, 160 to 180 as marked, and above 180 as excellent.

Bloody diarrhea in chickens was evaluated by daily observation under veterinary supervision and counting of bloody feces (count number of blood pieces present in feces after parasite oocyst penetrate the cecal part of chicken intestine via the fecal-oral route, and multiples in its epithelium) through the 4th to 7th d post-infection period. The chickens’ bloody diarrhea is one of the clinical signs of coccidiosis, and here considered one parameter to calculate the anti-coccidial index. In the morning, during the 4th to 7th day’s post-infection period, bloody chicken diarrhea was achieved by observing and counting the number of blood pieces present in the birds’ feces trays. After each observation, the feces were removed to see the new bloody pieces, and so on every day. The mean number of pieces of bloody feces in each treatment was assigned from 0 to 4 and rounded to the nearest integer. Briefly, 0 represented normal feces, 1 indicated one piece, 2 represented two pieces, 3 indicated three pieces, and so on, respectively. The fecal score percentage was then determined as (total highest fecal score for each division in PC minus total highest fecal score for each division in the medicated group)/total highest fecal score for each division in PC multiplied by 100.

The rate of inhibition of oocyst production was calculated as described by [45] as follows:

(1) $$\text{Inhibition rate}\;\left(\%\right)\;=\;\;\frac{\text{No. of oocysts per gram (OPG) produced by positive control (PC) group}\;-\;\text{No. of OPG produced by medicated infected group}}{\text{No. of OPG produced by PC group}}\times 100$$

(2) $$\mathrm{Oocyst}\;\mathrm{value}\;\left(\%\right)\;=\;\frac{\mathrm{No}.\;\mathrm{of}\;\mathrm{OPG}\;\mathrm{output}\;\mathrm{of}\;\mathrm{every}\;\mathrm{group}}{\mathrm{No}.\;\mathrm{of}\;\mathrm{OPG}\;\mathrm{output}\;\mathrm{of}\;\mathrm{PC}\;\mathrm{group}}\times 100$$

Excreta samples of each replicate were collected on the 7th d of infection from each pen. Each fecal sample was mixed thoroughly and dispersed in 10 mL of water. The number of oocysts in a suspension of 10 μL was then counted using a glass microscope slides and cleaned square microscope glass cover slides coverslips, 18 mm × 18mm under the Olympus optical microscope (Olympus Corporation, Tokyo, Japan) provided with microscope digital camera [46]. The results were expressed as OPG output.

Clinical signs and mortality were examined and recorded every day after infection. The survival rate was then calculated as the number of survived birds relative to the number of initial birds.

At the end of the trial (d 7 post-infection) at 28 d of age of the Ross 308 broiler chickens, a sample of 30 birds, with 1 bird/1 replicate, *n* = 5 birds in each group were subjected to feed withdrawal for 10 h, but drinking water was provided ad libitum during the feed withdrawal period. The birds then slaughtered and cut according to the law of animal welfare. After slaughtering by cutting the jugular vein, birds were left for complete bleeding and were then de-feathered and eviscerated. The cecum of each bird was removed and examined. The lesion scores ranged macroscopic lesions observed at necropsy on a scale of from 0 to 4 depending on the severity of the lesion and atrophy of the cecum. Where 0 refers to normal status and no gross lesions, 1 to small scattered petechiae, 2 to numerous petechiae, 3 to extensive hemorrhage, and 4 to extensive hemorrhage that provides a dark color to the cecal of the birds including coccidiosis-related death. Moreover, the length of the ceca was measured and recorded as mean ± SEM (in cm) to assess the degree of atrophy.

### 2.5. Performance Indices

To evaluate the bird’s performance measurements, all birds were weighed separately and the body weight gain (BWG) was calculated by subtracting the initial BW from the final BW (subtracting BW at 7 d postinfection from BW on the day birds were challenged with *Eimeria* oocysts). The feed consumption (FI) of birds in each pen was measured at the end of the test by subtracting the residual feed weight from the feed provided at the start of emeriosis challenge. Then, the feed conversion ratio was calculated as FCR = feed intake (g)/BWG (g) [47]. The European production efficiency factor (EPEF) was calculated as follows [27]:

(3) $$\mathrm{EPEF}\;=\frac{\mathrm{Livability}\times \mathrm{Live}\;\mathrm{weight}\;\left(\mathrm{Kg}\right)}{\mathrm{Age}\;\mathrm{in}\;\mathrm{days}\times \mathrm{FCR}}\times 100$$

The following formula was used to compare performance [48]:

(4) $$\mathrm{Performance}\;\mathrm{index}\;\left(\mathrm{PI}\right)\;=\frac{\mathrm{total}\;\mathrm{weight}\;\mathrm{gain}}{\mathrm{total}\;\mathrm{feed}\;\mathrm{conversion}}\times 100$$

### 2.6. Statistical Analysis

All data were statistically analyzed using All data were statistically analyzed using general linear models procedure of SAS (Statistical Analysis System) software (SAS, 2012, SAS Institute Inc., Cary, NC, USA) [49]. All data were subjected to one-way analysis of variance and expressed as mean ± SEM.

For the analysis of variables, the following statistical ANOVA models were used:

Yij = μ + Ti + eij

where: Yij = the observed j variable in the ith treatment; μ = overall mean of the variables; Ti = the effect the ith treatment; and eij = random residual error. For statistical differences, all mean values were separated using the Ryan–Einot–Gabriel–Welsch multiple range.

## 3. Results

### 3.1. Nutrient Analysis and Phytochemical Composition of RN Leaves

**Figure 1:**
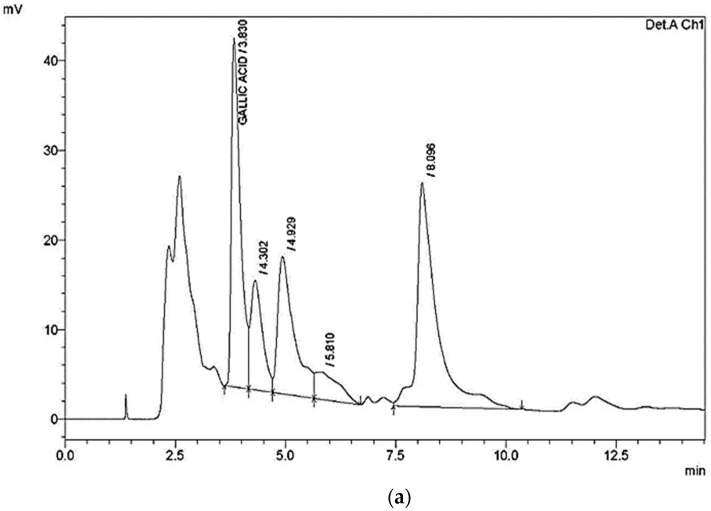

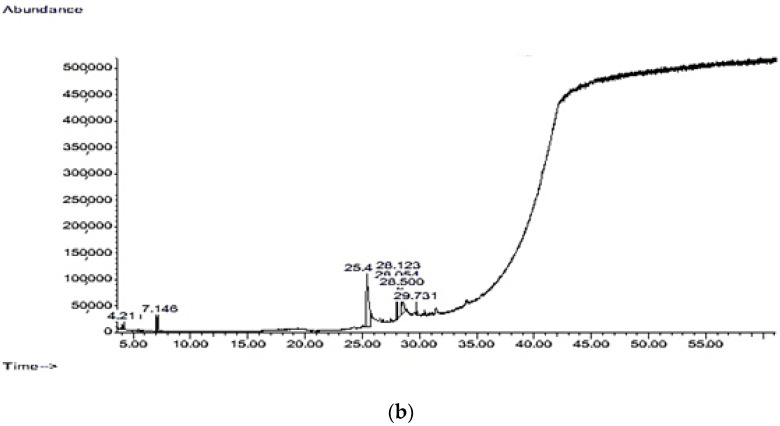
(**a**) HPLC chromatogram of the standard mixture of flavonoids and phenols of *Rumex nervosus* at 280 nm; (**b**) Gas chromatography-mass spectrometry (GC-MS) tracing of *Rumex nervosus* leaf extract.

The chemical composition of RN leaves is shown in Table 2. The crude protein, crude fiber, ash, neutral detergent fiber, acid detergent fiber, and energy were detected at 13.63%, 8.24%, 18.01%, 20.21%, 15.48%, and 3273.31 kcal/kg, respectively, which indicated its potential as a nutraceutical. Gallic acid was the component detected with the largest composition (700 µg/g) in RN leaf extracts based on the HPLC results (Table 3; Figure 1a). GC-MS was used to analyze the leaf extracts of RN, which identified the compounds shown in Table 4 and Figure 1b. Fatty acids were found to be the most abundant compounds.

### 3.2. Anti-Coccidial Activity

**Figure 2:**
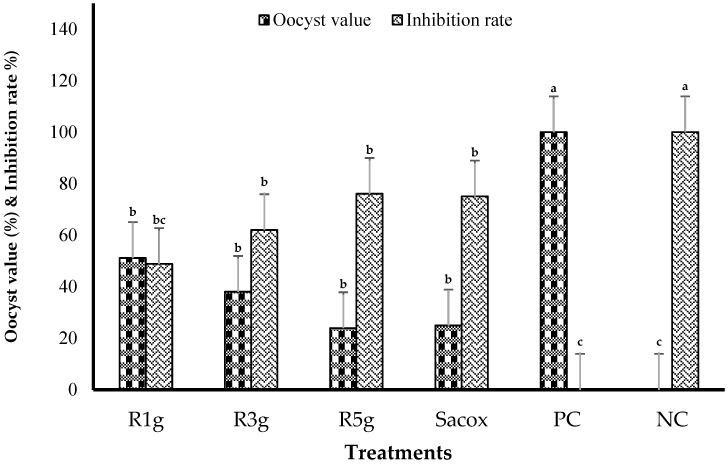
Effect of experimental treatments and challenge with *E. tenella* on oocyst value and inhibition rate at 7 d postinoculation; R1g, R3g, and R5g are 1, 3, and 5 g RN/kg diet, respectively, Sacox: 66 mg of salinomycin/kg diet; PC and NC: basal diet with and without coccidiosis challenge, respectively. Different lowercase letters indicate significant differences.

**Figure 3:**
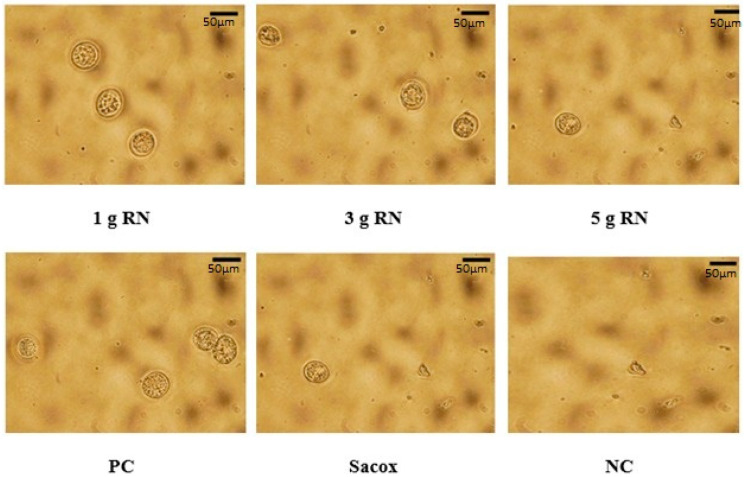
Oocyst morphology of *E. tenella* in the infected experimental treatments. R1g, R3g, and R5g are 1, 3, and 5 g RN/kg diet, respectively, Sacox: 66 mg of salinomycin/kg diet, PC and NC: basal diet with and without coccidiosis challenge, respectively. Magnification under object lens 40× and scale bar 50 µm.

Clinical signs of coccidia parasite infection such as huddling together, ruffled feathers, and depression were detected in the infected birds but not in the uninfected birds. Table 5 shows the effects of RN leaf powder on the mean output of OPG, lesion score, oocyst value, bloody diarrhea, inhibition rate, and cecal length of each experimental group at d 7. There was no mortality in all the experimental groups, except for one bird in the PC group (group 5). Bloody diarrhea and fecal output of oocysts were absent during the first 4 d after the challenge. Bloody diarrhea was observed in groups 1 to 5 from the 4th to 7th d after *E. tenella* challenge. The dietary supplementation of 5 g/kg RN reduced the OPG, oocyst value, lesion score, bloody diarrhea, and cecal length atrophy of broilers at the 7th d after infection, whereas treatment with 1 g/kg RN produced the opposite results; however, the differences were not significant (*p* > 0.05). Therefore, after feeding with RN leaf powder, the anti-coccidial activity among the birds appeared to be dose-dependent but not significant when compared with each other (*p* > 0.05). Furthermore, the anti-coccidial parameters of birds in groups fed with RN-medicated diets at the dose used in this study were not significant compared to those evaluated after Sacox treatment. Moreover, the birds’ OPG, oocyst value, lesion score, bloody diarrhea, and cecal length atrophy in RN- and Sacox-medicated groups were significantly reduced (*p* < 0.05) compared with birds in the PC group. Consequently, severe pathological features such as higher OPG, oocyst value, lesion score, bloody diarrhea, and cecal length atrophy were noticed in the PC group (*p* < 0.05) compared to those in the NC group. The inhibition rates of RN-medicated groups 1, 2, and 3 and Sacox-medicated group were 48.85%, 61.99%, and 76.07% and 75.06%, respectively (Figure 2). A relationship was observed in which oocyst output reduced with increasing extract dose. Oocyst morphology of *E. tenella* in the infected experimental treatments is shown in Figure 3.

**Figure 4:**
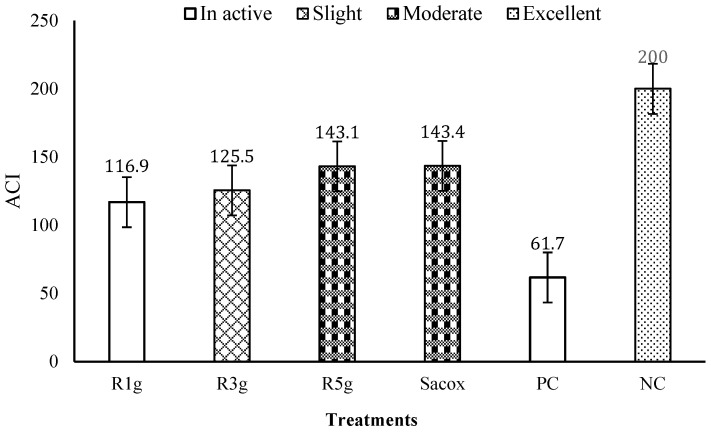
Effect of experimental treatments and coccidiosis challenge on the anti-coccidial index (ACI) of each group at 7 d postinoculation. R1g, R3g, and R5g represent 1, 3, and 5 g RN/kg diet, respectively, Sacox: 66 mg salinomycin/kg diet, PC and NC: basal diet with and without coccidiosis challenge, respectively. The ACI value below 120 indicated an inactive anti-coccidial effect, 120 to 140 as mild or slight, 140 to 160 as moderate, 160 to 180 as marked, and above 180 as excellent.

As shown in Figure 4, the PC group showed the lowest ACI value (62). The RN-medicated groups 1, 2, and 3 showed inactive, slight, and intermediate anti-coccidial impacts, with ACI indexes of 117, 125, and 143, respectively, and the ACI index of the infected group supplemented with Sacox reached 143, indicating an intermediate anti-coccidial effect. The PC group exhibited a higher ACI value (200) than the infected group supplemented with Sacox.

**Figure 5:**
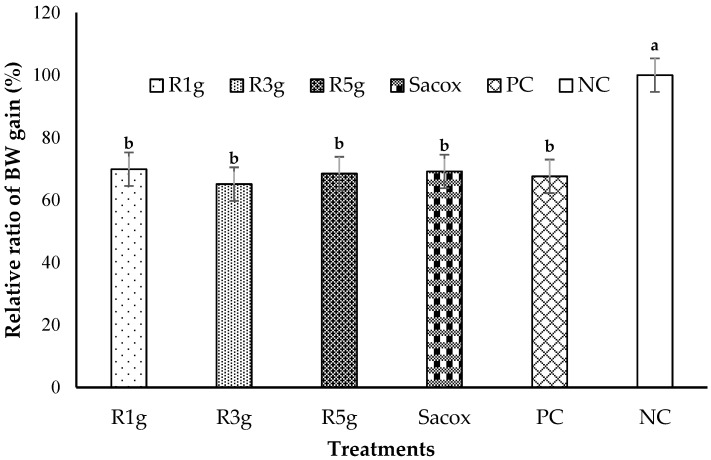
Effect of experimental treatments and infection with *E. tenella* on the relative ratio of body weight gain of birds at 7 d postinoculation. (R1g, R3g, and R5g represent 2, 4, and 6 g RN/kg diet, respectively, Sacox: 66 mg salinomycin/kg diet, PC and NC: basal diet with and without coccidiosis challenge, respectively. Different lowercase letters indicate significant differences.

The mean relative BWG ratio of each group is shown in Figure 5. The initial weights between groups of chickens were not significant (data are not shown). The BW of all three RN-supplemented groups did not increase significantly when compared between groups or with the PC group (*p* > 0.05) or even with the Sacox-treated group. Therefore, our data demonstrated that the RN leaf powder did not increase the BWG effectively at the current dose used.

### 3.3. Performance and Production Efficiency

Table 6 shows the effects of RN leaf powder on the feed efficiency of broilers. Dietary supplementation of 5 g/kg RN increased the BW, FI, production efficiency factor (PEF) of broilers on the 7th d after infection, whereas treatment with 3 g/kg RN reduced the BWG, FI, and PEF, but with no significant differences (*p* > 0.05). Feeding with RN leaf powder produced no significant differences in the performance parameters of birds (*p* > 0.05). Moreover, the performance parameters of birds in the RN-medicated groups at the current dose were not significant compared to those observed in the Sacox-treated or PC groups.

Moreover, the BW, FI, FCR, and PEF of birds in the RN-medicated groups were significantly lower (*p* < 0.05) than in the NC group. Birds in the NC group gained excessive body weight, consumed excessive feed, and converted additional competent feed as a result of increased FEF compared with birds in the NC group.

## 4. Discussion

Salinomycin (Sacox^®^) anti-coccidial drugs are developed from natural products and broadly used in the poultry sector for controlling coccidiosis. We attempted to mimic a synthetic anti-coccidial drug to evaluate a natural herb with anti-coccidial properties that could eventually be further developed into a new, safe, anti-coccidial drug without tissue residues or even drug resistance. After several herbal plant surveys, we focused on the RN leaf as there are no or only a few scientific articles addressing the impact of dietary supplementation of RN leaf powder in the broiler diet. This shrub is rich in several chemical ingredients such as carbohydrates, sterols, glycosides, triterpenes, saponins, tannins, and flavonoids in its flowers [50] and root, stems, and leaves [30]. These diverse phytochemicals bestow this shrub with various bioactivities. Various environmental factors may, of course, alter the quantitative and qualitative aspects of essential oils (EO) in a variety of ways, such as altering the metabolism of plants and their secondary metabolites. The chemical composition of EO depends on climatic, seasonal, and geographical conditions and harvesting age, distillation technique, storage conditions, the origin of the plant, part of the plant used, and the stage of development and growth of the habitat (soil, temperature, and fertilizers). Moreover, their anti-microbial activity depends on the type of composition and concentration of the EO, the type and concentration of the target microorganism, the composition of the substrate, and the processing and storage conditions [51,52]. However, the effect of environmental factors on the chemical composition and yield of compounds that are believed to help control coccidia is still unknown and needs to be investigated in the future.

Coccidiosis is among the most dangerous infectious disease known as red dysentery caused by the destruction of intestinal epithelial cells, resulting in severe bloody diarrhea in poultry. Our study results are consistent with those reported by [44] who observed similar and typical signs of chicken coccidiosis, such as bloody diarrhea, ruffled feathers, wing drooping, and other signs of infection.

Our data demonstrated that RN leaf powder supplementation considerably reduced bloody diarrhea in the challenged birds. A reduction in blood loss can help defend the infected broilers from secondary bacterial invasion, inflammatory reactions, and absorption of toxic compounds [53]. The anti-diarrheal activity of RN may be attributed to therapeutic effects such as intestinal motility reduction and direct anti-coccidial activity. It may be due to the fact that RN contains a higher amount of the flavonoid compound “quercetin”, which hinders the release of acetylcholine into the digestive tract, an assumption supported by similar claims made by [54].

The amount of oocysts in feces is a crucial indicator of the spread of coccidiosis through modern intensive poultry farming due to the transmission of *Eimeria* oocysts via the oral-fecal route [55]. Consistent with previous reports [27,44,56,57], the number of fecal oocysts in chickens challenged with coccidia were effectively reduced in response to the natural product. The RN leaf powder dramatically reduced the OPG output in the RN-treated groups, suggesting that RN leaf powder could contribute to the control of coccidiosis outbreaks in large-scale poultry farms.

The cecum is among the most influential digestive organs in broilers. Infection with *Eimeria* could cause major epithelial destruction. Therefore, the host may sustain diarrhea and malabsorption related to poor weight gain [58,59]. Gross lesion score [60] and cecum atrophy varied from mild atrophy and scattered petechiae in the RN-medicated groups to serious atrophy and severe hemorrhage in the PC group. The GLS of the cecum of the birds was reduced mostly in the Sacox-treated group. These results demonstrated that bioactive ingredients present in RN leaf powder supplementation can have indirect anti-coccidial action and can play a role in the control of eimeriosis in birds by reducing the lesion score, oocysts output, suppressing or delaying the developmental *E. tenella* parasite, and mortality. Therefore, RN leaf powder can play a vital role in developing the organ-protective properties of infected birds [61].

The damaging effect of coccidiosis was obvious in the challenged group that was ameliorated treatment with the RN herb. The positive impact of RN leaf extract in terms of the anti-coccidial criterion can be attributed to the presence of abundant flavonoids, alkaloids, tannins, and saponins, which may exert a combined effect of anti-parasitic, anti-inflammatory, and anti-diarrheal activity in birds [62,63]. In the present study, the chemical composition of RN leaves methanolic extracts was studied using HPLC and GC-MS analysis and resulted in the identification of Gallic acid and 13 compounds, respectively. Further studies are needed to investigate the anti-coccidial potential of active ingredients either alone or in mixtures with other anti-coccidial substances. Gallic acid is a phenolic acid widely distributed in plants and foods and has a wide range of activities, including anti-oxidant, anti-inflammatory, anti-bacterial, anti-allergic, anti-carcinogenic, and ant-mutagenic properties. Therefore, natural or synthetic anti-oxidants are commonly used as feed supplements in the poultry industry to relieve or reduce oxidative stress caused by the high production of free radical oxidative species during the host cell immune response to *Eimeria* invasion [64]. This can play an important role in the defense against parasitic infections and in the scavenging of tissue damage and cytotoxicity. A total of 13 compounds were detected in the GC-MS analysis of the methanolic extract of RN leaf, whose activity and mode of action against coccidiosis must be investigated in further studies.

Our study findings are in agreement with those observed by [65] who reported that natural herbal components and their extracts such as myrobalan, *Quercus infectoria*, *Rhus chinensis*, and *Quercus infectoria*, containing gallic acid, gallotannins, tannins, and other compounds, play a role in controlling eimeriosis in birds, such as reduction of lesion score, oocyst output, and mortality.

As anticipated, the performance in the PC group was most adversely affected. These results are consistent with those of [66] who showed that *Eimeria*-infected birds exhibited a significant reduction of body weight and feed consumption compared with uninfected birds due to coccidiosis infection, resulting in poor nutrient absorption and decreased immune response and consequently intestinal tissue injury [67]. However, the results indicated that the BW, FCR, PI, and the production efficacy of the RN- and Sacox-treated groups were negatively affected (*p* < 0.05) by the coccidia challenge compared to those in the NC group that resulted in reduced weight gain due to coccidiosis. Fortunately, the performance parameters were positively affected in the RN- and Sacox-treated groups compared to those in the PC group, which are in agreement with the results reported by [27]; however, the present study values were not significant. The non-significant findings observed in both groups in this study were incompatible with those reported by [27] who reported high values for BWG, BW, and PEF in the group treated with a synthetic anti-coccidial drug (Elancoban). In the present study, the performance was not dependent on the dose. In contrast to the result of [57], it was observed that the performance increased linearly with an increase in dose, excluding feed consumption. The synthetic drug Sacox used as a control in this study had an intermediate anti-coccidial effect. Here, the birds in the Sacox treated group were consumed with higher feed compared to the birds in other infected groups, resulting in the worst feed conversion and feed efficiency due to the reduced gain and higher feed intake of birds. Our findings differ from those of [12,13] which found that Sacox was the best performing groups.

RN and even Sacox groups were lower BW and BWG compared to NC, which resulted in reduced weight gain due to coccidiosis. The birds had a higher body weight in the Sacox groups than the PC; however, gain did not differ.

Besides the above mentioned important findings that strengthen and support the present study results represent in a reduction of Oocyst number and lesions in the cecum, there are some limitations. For instance, although the data on the performance index, weight gain, and production efficiency do not appear to support this shrub in promoting performance, it is likely that challenged birds by an overdose of coccidian oocysts or an ineffective dose of RN leaf powder supplementation (such as 1g RN/kg diet) have an undesirable effect on the chickens. Therefore, further studies must be conducted carefully using a lower dose of emeriosis oocysts or a higher medicinal dosage to relieve the undesirable effects on BW. Furthermore, in order to evaluate an in vitro anti-coccidial efficacy of RN leaf, it is necessary to, determine lethal dose 50 (LD_50_) of RN leaf extract, isolate and identify pure components to understand the relationship between the major components and their corresponding anti-coccidial effects to allow new drug discovery. In the present study, our data confirmed that the RN leaf powder yields intermediate anti-coccidial effects. In this study, the nutritional proximate analysis revealed that RN had a moderate raw protein content (13.63 percent) and a high carbohydrate content (52.91 percent) resulted in a higher nutritional value (327.66 Kcal/100 g). There was no harmful effect of currently used anti-coccidial drugs to broilers or even to human based on pharmacognostical study of RN growing in Yemen reported by [30], who found that the ethanolic extract (70%) of RN considered as non-toxic up to a dose of 7.1 g/kg b.wt and have cytotoxic activity with varying degrees of potency against human cancer cell lines. We suggest that RN in the range of doses administered could be considered non-toxic.

Although our data have the intermediate activity of RN against coccidia with higher dose, the performance parameters were higher numerical with lower dose at the same time. Thus, both sides take into account the anti-cocidal and improvement performance of RN for future prescript. As a result, our results suggested that the dose could be increased by 5 g RN or more with good expected result on coccidiosis oocysts shedding when added to the broiler diet or used as an extract for further improvement, if possible, in order to increase the efficacy of RN. However, further studies are needed for its application, perhaps alone or with other herbal products, which could improve the feed conversion rate, increase body weight gain, and reduce coccidia oocysts together.

## 5. Conclusions

In conclusion, dietary RN leaf powder supplementation indicated that RN powder at level 5 g RN/kg diet possesses moderate anti-coccidial effects through reduced the lesion scores, suppressed the output of oocysts per gram in chickens’ feces, and hence could be used to treat avian coccidiosis in field conditions. Although RN was unable to minimize the weight gain loss caused by infection with emeriosis, RN leaf powder at level 1 g RN/kg diet improved feed conversion ratio and attempted to improved body weight gain, performance index, and production efficiency at 7-days post-infection compared to the infected control group. In addition, the wide distribution, economic nature, and simplicity of using the Ithrib shrub suggested that RN leaf powder could also act as a moderate alternative to anti-coccidial agents. This study would eventually support a novel strategy for controlling broiler chicken coccidiosis in field conditions.

## Figures and Tables

**Table 1:** Ingredients and calculated nutrients of broiler starter and finisher diets.

Ingredient	Period	
	Starter	Finisher
Yellow corn	53.218	58.09
Soybean meal	37.85	32.15
Wheat bran	2.00	2.2
Corn gluten meal	1.4	0
Choline chloride CL 60	0.05	0.05
Corn oil	1.5	4.2
Dicalcuim phosphate DCP	1.98	1.615
Ground limestone	0.9	0.79
Salt	0.400	0.30
DL-methionine	0.292	0.25
Lysine-HCL	0.21	0.105
Vitamin–mineral premix ^1^	0.200	0.200
Total	100	100
Metabolic energy (ME), kcal/kg	3000	3200
Crude protein, %	23.0	20.0
Non phytate *p*, %	0.48	0.405
Calcium, %	0.96	0.81
d-lysine, %	1.28	1.06
Sulfur amino acids, %	0.95	0.83
Threonine, %	0.86	0.71

^1^ Vitamin–mineral premix contains the following per kg: vitamin A, 12,000,000 IU; vitamin D3, 5,000,000 IU; vitamin E, 80,000 IU; vitamin K3, 3200 mg; vitamin B_1_, 3200 mg; vitamin B_2_, 8600 mg; vitamin B_3_, 65,000 mg; pantothenic acid, 20,000 mg; vitamin B_6_, 4300 mg; biotin 220 mg; anti-oxidant (BHA+BHT), 50,000 mg; B_9_, 2200 mg; B_12_, 17 mg; copper, 16,000 mg; iodine, 1250 mg; iron, 20,000 mg; manganese, 120,000 mg; selenium, 300 mg, and zinc, 110,000 mg.

**Table 2:** Proximate composition of dried leaves of *Rumex nervosus* ^1^.

Parameter	As-fed basis (%)
Moisture content	5.67
Dry matter	94.33
Crude protein	13.63
Ether extract	1.54
NFE	58.58
Total carbohydrate	52.91
Crude ash (Inorganic matter)	18.01
Total crude fiber	8.24
Total fiber fractions	
A. Acid detergent fiber	15.48
B. Neutral detergent fiber	20.21
Organic matter	81.99
Nutritive value (GE) (kcal/100 g)	327.33

^1^ Values are the results of a chemical analysis of three replicates; GE: gross energy; NFE: nitrogen-free extract or digestible carbohydrates (calculated by difference) = 100 − (protein + lipid + ash + fiber); Total carbohydrate = 100 − (% ash + % moisture + % crude fiber + % crude protein); Organic matter = 100 − Ash

**Table 3:** Contents of individual flavonoids and phenols (µg/g) of *Rumex nervosus* determined by HPLC.

Compound	Retention Time (RT) (min)	Area	Height	Concentration (µg/g)
Gallic acid	3.830	554,308	38,945	700
Catechin	-	-	-	-
Chlorogenic acid	-	-	-	-
Caffeine	-	-	-	-

**Table 4:** Main compounds (%) detected by gas chromatography-mass spectrometry (GC-MS) in the methanolic extract of *Rumex nervosus* leaf.

Retention Time (RT) (min)	Bioactive Chemical Constituents	Quality	Molecular Weight (amu)	Molecular Formula
4.211	Oxime-, methoxy-phenyl-_	74	151.063	C_8_H_9_NO_2_
7.026	Cyclotrisiloxane, hexamethyl-	72	222.056	C_6_H_18_O_3_Si_3_
7.146	5-Methyl-2-phenylindolizine	59	207.105	C_15_H_13_N
7.146	1,2-Bis(trimethylsilyl)benzene	50	222.126	C_12_H_22_Si_2_
25.41	Hexadecanoic acid, methyl ester	98	270.256	C_15_H_30_O_2_
25.41	Pentadecanoic acid, 14-methyl-, methyl ester	97	270.256	C_17_H_34_O_2_
25.41	Tridecanoic acid, methyl ester	86	228.209	C_14_H_28_O_2_
25.41	Nonadecanoic acid, methyl ester	53	312.303	C_20_H_40_O_2_
28.054	9,12-Octadecadienoic acid (Z,Z)-, methyl ester	99	294.256	C_19_H_34_O_2_
28.054	9,17-Octadecadienal, (Z)-	90	264.245	C_18_H_32_O
28.123	10,13-Octadecadienoic acid, methyl ester	99	294.256	C_19_H_34_O_2_
28.123	7-Pentadecyne	96	208.219	C_15_H_28_
28.5	Octadecanoic acid, methyl ester	98	298.287	C_19_H_38_O_2_

**Table 5:** Effect of different experimental supplements and challenge on oocyst/gram (OPG) output, lesion score, oocyst value, inhibition rate, and cecal length at 7 d postinoculation.

Group	Treatment ^1^	Bloody Diarrhea	Lesion Scores	OPG (Mean × 10^6^)	Oocyst Value	Inhibition Rate	Cecal Length (%)
1	RN1g	1.0 ^ab^	1.8 ^ab^	6.42 ^b^	51.15 ^b^	48.85 ^b^	15.56 ^ab^
2	RN3g	0.8 ^ab^	1.6 ^b^	4.77 ^bc^	38.01 ^bc^	61.99 ^b^	16.83 ^a^
3	RN5g	0.2 ^b^	1.4 ^b^	3.00 ^bc^	23.93 ^bc^	76.07 ^b^	16.44 ^a^
4	Sacox	0.0 ^b^	0.8 ^bc^	3.13 ^bc^	24.94 ^bc^	75.06 ^b^	15.88 ^ab^
5	PC	2.0 ^a^	2.6 ^a^	12.54 ^a^	100.00 ^a^	0.00 ^c^	11.99 ^b^
6	NC	0.0 ^b^	0.0 ^c^	0.00 ^c^	0.00^c^	100.00 ^a^	14.79 ^ab^
SEM ^2^ Probability		0.205	0.176	0.8796	7.012	7.454	0.459
		0.0196	<0.0001	0.0010	<0.0001	0.0010	0.0180

^1^ RN1g, RN3g, and RN5g = Infected supplemented with 1, 3, and 5 g *Rumex nervosus* leaf powder/kg diet, respectively; Sacox = Infected supplemented with Sacox (66 mg/kg diet); PC = Infected untreated control, NC = Uninfected untreated control; ^2^ SEM: Standard error means; ^a–c^ Columns with different letters indicate statistically significant differences (*p* < 0.05).

**Table 6:** Live body weight (BW), live body gain (BWG), cumulative feed intake (FI), feed conversion ratio (FCR), performance index (PI), and production efficiency factor (PEF) of birds fed with experimental diets.

Group	Treatment ^1^		Performance				
		BW (kg)	BWG (g)	FI (g)	FCR (g:g)	PI	PEF
1	R1g	1.128 ^b^	58.35 ^b^	96.87 ^b^	1.67 ^ab^	35.36 ^b^	251.90 ^b^
2	R3g	1.136 ^b^	54.37 ^b^	96.45 ^b^	1.77 ^a^	30.71 ^b^	237.49 ^b^
3	R5g	1.200 ^ab^	57.18 ^b^	98.80 ^b^	1.74 ^a^	33.29 ^b^	257.61 ^b^
4	Sacox	1.238 ^ab^	57.75 ^b^	104.35 ^ab^	1.81 ^a^	32.03 ^b^	254.19 ^b^
5	PC	1.120 ^b^	56.45 ^b^	99.35 ^b^	1.81 ^a^	33.14 ^b^	230.64 ^b^
6	NC	1.273 ^a^	83.51 ^a^	117.19 ^a^	1.41 ^b^	59.76 ^a^	336.18 ^a^
SEM ^2^ Probability		18.082	2.272	1.950	0.040	2.313	9.068
		0.043	<0.0001	0.007	0.017	<.0001	0.003

^1^ RN1g, RN3g, and RN5g = Infected supplemented with 1, 3, and 5 g *Rumex nervosus* leaf powder/kg diet, respectively; Sacox = Infected supplemented with Sacox (66 mg/kg diet); PC = Infected untreated control, NC = uninfected untreated control; ^2^ SEM: Standard error means; ^a–c^ Columns with different letters indicate statistically significant differences (*p* < 0.05).

## Data Availability

All data sets collected and analyzed during the current study are available from the corresponding author on fair request.

## References

[1] Peek H., Landman W. (2011). Coccidiosis in poultry: Anticoccidial products, vaccines and other prevention strategies. Vet. Q..

[2] Taylor M., Coop R., Wall R. (2007). Veterinary Parasitology.

[3] Michels M., Bertolini L., Esteves A., Moreira P., Franca S. (2011). Anticoccidial effects of coumestans from Eclipta alba for sustainable control of Eimeria tenella parasitosis in poultry production. Vet. Parasitol..

[4] Abdel-Baki A.-A.S., Al-Quraishy S. (2013). Prevalence of Coccidia (*Eimeria* spp.) Infection in Domestic Rabbits, Oryctolagus cuniculus, in Riyadh, Saudi Arabia. Pak. J. Zool..

[5] Al-Quraishy S., Abdel-Baki A., Dkhil M. (2009). Eimeria tenella infection among broiler chicks Gallus domesticus in Riyadh city, Saudi Arabia. J. King Saud Univ. Sci..

[6] Dubey J.P. (2019). Coccidiosis in Livestock, Poultry, Companion Animals and Humans.

[7] Kim D.K., Lillehoj H.S., Lee S.H., Jang S.I., Lillehoj E.P., Bravo D. (2013). Dietary Curcuma longa enhances resistance against Eimeria maxima and Eimeria tenella infections in chickens. Poult. Sci..

[8] Amerah A., Ravindran V. (2015). Effect of coccidia challenge and natural betaine supplementation on performance, nutrient utilization, and intestinal lesion scores of broiler chickens fed suboptimal level of dietary methionine. Poult. Sci..

[9] Fang Z., Liu W., Shi P., Zhang Y., Huang Z. (2016). Protective effect of berberine on the intestinal caecum in chicks with Eimeria tenella. Avian Biol. Res..

[10] Upadhaya S.D., Cho S.H., Chung T.K., Kim I.H. (2019). Anti-coccidial effect of essential oil blends and vitamin D on broiler chickens vaccinated with purified mixture of coccidian oocyst from Eimeria tenella and Eimeria maxima. Poult. Sci..

[11] Nouroozikoh T., Shirali S., Rahbari S. (2018). Salinomycin Impact on Eimeria Species Oocyst Excretion in Poultry by Litter Monitoring. Vet. Res. Biol. Prod..

[12] Abdelrahman W., Mohnl M., Teichmann K., Doupovec B., Schatzmayr G., Lumpkins B., Mathis G. (2014). Comparative evaluation of probiotic and salinomycin effects on performance and coccidiosis control in broiler chickens. Poult. Sci..

[13] Tonda R., Rubach J., Lumpkins B., Mathis G., Poss M. (2018). Effects of tannic acid extract on performance and intestinal health of broiler chickens following coccidiosis vaccination and/or a mixed-species Eimeria challenge. Poult. Sci..

[14] Robinson K., Becker S., Xiao Y., Lyu W., Yang Q., Zhu H., Yang H., Zhao J., Zhang G. (2019). Differential impact of subtherapeutic antibiotics and ionophores on intestinal microbiota of broilers. Microorganisms.

[15] Abbas R., Iqbal Z., Blake D., Khan M., Saleemi M. (2011). Anticoccidial drug resistance in fowl coccidia: The state of play revisited. Worlds Poult. Sci. J..

[16] Wunderlich F., Al-Quraishy S., Steinbrenner H., Sies H., Dkhil M.A. (2014). Towards identifying novel anti-Eimeria agents: Trace elements, vitamins, and plant-based natural products. Parasitol. Res..

[17] Barbour E., Ayyash D., Iyer A., Harakeh S., Kumosani T. (2015). A review of approaches targeting the replacement of coccidiostat application in poultry production. Rev. Bras. Cienc..

[18] Wiedosari E., Wardhana A.H. (2018). Anticoccidial activity of Artemisinin and Extract of Artemesia annua leaves in chicken infected by Eimeria tenella. Ind. J. Anim. Vet. Sci..

[19] Abbas A., Iqbal Z., Abbas R.Z., Khan M.K., Khan J.A., Mahmood M.S., Saleemi M.K. (2017). In vivo anticoccidial effects of Beta vulgaris (sugar beet) in broiler chickens. Microb. Pathog..

[20] Allen P.C. (2003). Dietary supplementation with Echinacea and development of immunity to challenge infection with coccidia. Parasitol. Res..

[21] Yim D., Kang S.S., Kim D.W., Kim S.H., Lillehoj H.S., Min W. (2011). Protective effects of Aloe vera-based diets in Eimeria maxima-infected broiler chickens. Exp. Parasitol..

[22] Toulah F., Ismeel H., Khan S. (2010). Effect of treatment with Neem (Azadirachta indica) compared with Baycox drug on the caecum of chicken experimentally infected with Eimeria tenella. J. Egypt. Soc. Parasitol..

[23] Ullah M.I., Akhtar M., Awais M.M., Anwar M.I., Khaliq K. (2018). Evaluation of immunostimulatory and immunotherapeutic effects of tropical mushroom (*Lentinus edodes*) against eimeriasis in chicken. Trop. Anim. Health Prod..

[24] Abbas R., Colwell D., Gilleard J. (2012). Botanicals: An alternative approach for the control of avian coccidiosis. Worlds Poult. Sci. J..

[25] Quiroz-Castañeda R.E., Dantán-González E. (2015). Control of avian coccidiosis: Future and present natural alternatives. Biomed. Res. Int..

[26] Al-Naqeb G. (2015). Antioxidant and antibacterial activities of some Yemeni medicinal plants. Int. J. Herb. Med..

[27] Alhotan R.A., Abudabos A. (2019). Anticoccidial and antioxidant effects of plants derived polyphenol in broilers exposed to induced coccidiosis. Environ. Sci. Pollut. Res..

[28] Goodla L., Manubolu M., Pathakoti K., Jayakumar T., Sheu J.-R., Fraker M., Tchounwou P.B., Poondamalli P.R. (2019). Protective effects of ammannia baccifera against CCl4-induced oxidative stress in rats. Int. J. Environ. Res..

[29] Al-Asmari A.R.K., Siddiqui Y.M., Athar M.T., Al-Buraidi A., Al-Eid A., Horaib G.B. (2015). Antimicrobial activity of aqueous and organic extracts of a Saudi medicinal plant: Rumex nervosus. J. Pharm. Bioallied Sci..

[30] Al-Sunafi S.M.Y. (2016). Pharmacognostical Study of Rumex nervosus Vahl. Family (Polygonaceae) growing in Yemen. Master’s Thesis.

[31] Shankar P.R., Balasubramanium R. (2014). Antimicrobial resistance: Global report on surveillance 2014. Australas. Med. J..

[32] Desta K.T., Lee W.S., Lee S.J., Kim Y.H., Kim G.S., Lee S.J., Kim S.T., Abd El-Aty A., Warda M., Shin H.C. (2016). Antioxidant activities and liquid chromatography with electrospray ionization tandem mass spectrometry characterization and quantification of the polyphenolic contents of Rumex nervosus Vahl leaves and stems. J. Sep. Sci..

[33] Quradha M.M., Khan R., Rehman M.U., Abohajeb A. (2019). Chemical composition and in vitro anticancer, antimicrobial and antioxidant activities of essential oil and methanol extract from Rumex nervosus. Nat. Prod. Res..

[34] Qasem M.A., Dkhil M.A., Al-Shaebi E.M., Murshed M., Mares M., Al-Quraishy S. (2020). Rumex nervosus leaf extracts enhance the regulation of goblet cells and the inflammatory response during infection of chickens with Eimeria tenella. J. King Saud Univ. Sci..

[35] Al-Quraishy S., Qasem M.A., Al-Shaebi E.M., Murshed M., Mares M.M., Dkhil M.A. (2020). Rumex nervosus changed the oxidative status of chicken caecum infected with Eimeria tenella. J. King Saud Univ. Sci..

[36] Ali A., Nasser A., Al-Sokari S.S., Mothana R., Hamed M., Waigh M., Cos P., Maes L. (2016). In vitro antiprotozoal activity of five plant extracts from Albaha region. World J. Pharm. Res..

[37] Limenih Y., Umer S., Wolde-Mariam M. (2015). Ethnobotanical study on traditional medicinal plants in Dega Damot woreda, Amhara Region, North Ethiopia. Int. J. Res. Pharm. Chem..

[38] Vasas A., Orbán-Gyapai O., Hohmann J. (2015). The Genus Rumex: Review of traditional uses, phytochemistry and pharmacology. J. Ethnopharmacol..

[39] El-Ashram S., Suo X. (2017). Electrical cream separator coupled with vacuum filtration for the purification of eimerian oocysts and trichostrongylid eggs. Sci. Rep..

[40] Lee H.-A., Hong S., Chung Y.-H., Song K.-D., Kim O. (2012). Anticoccidial effects of Galla rhois extract on Eimeria tenella-infected chicken. Lab. Anim. Res..

[41] Melesse A., Masebo M., Abebe A. (2018). The Substitution Effect of Noug Seed (Guizotia Abyssinica) Cake with Cassava Leaf (Manihot Escutulata C.) Meal on Feed Intake, Growth Performance, and Carcass Traits in Broiler Chickens. J. Anim. Hus. Dairy Sci..

[42] Adaszyńska-Skwirzyńska M., Szczerbińska D. (2018). The effect of lavender (*Lavandula angustifolia*) essential oil as a drinking water supplement on the production performance, blood biochemical parameters, and ileal microflora in broiler chickens. Poult. Sci..

[43] Ma D., Ma C., Pan L., Li G., Yang J., Hong J., Cai H., Ren X. (2011). Vaccination of chickens with DNA vaccine encoding Eimeria acervulina 3-1E and chicken IL-15 offers protection against homologous challenge. Exp. Parasitol..

[44] Lan L., Zuo B., Ding H., Huang Y., Chen X., Du A. (2016). Anticoccidial evaluation of a traditional chinese medicine—Brucea javanica—in broilers. Poult. Sci..

[45] Chauhan S., Singh V., Thakur V. (2017). Effect of Calotropis procera (madar) and amprolium supplementation on parasitological parameters of broilers during mixed Eimeria species infection. Vet. World..

[46] Holdsworth P., Conway D., McKenzie M., Dayton A., Chapman H., Mathis G., Skinner J., Mundt H.-C., Williams R. (2004). World Association for the Advancement of Veterinary Parasitology (WAAVP) guidelines for evaluating the efficacy of anticoccidial drugs in chickens and turkeys. Vet. Parasitol..

[47] Panda A., Raju M., Rao S., Lavanya G., Reddy E., Sunder G.S. (2010). Replacement of normal maize with quality protein maize on performance, immune response and carcass characteristics of broiler chickens. Asian Australas. J. Anim. Sci..

[48] Behnamifar A., Rahimi S., Kiaei M., Fayazi H. (2019). Comparison of the effect of probiotic, prebiotic, salinomycin and vaccine in control of coccidiosis in broiler chickens. Iran. J. Vet. Res..

[49] SAS (2012). SAS Institute/OR 9.3 User’s Guide: Mathematical Programming Examples.

[50] Desta K.T., Kim G.S., Hong G.E., Kim Y.H., Lee W.S., Lee S.J., Jin J.S., Abd El-Aty A., Shin H.C., Shim J.H. (2015). Dietary-flavonoid-rich flowers of Rumex nervosus Vahl: Liquid chromatography with electrospray ionization tandem mass spectrometry profiling and in vitro anti-inflammatory effects. J. Sep. Sci..

[51] Ariza-Nieto C.J. (2006). Evaluation of Oregano (Origanum Vulgare) Essential Oils in Swine Production System.

[52] Castelo A., Del Menezzi C., Resck I. (2012). Seasonal variation in the yield and the chemical composition of essential oils from two Brazilian native arbustive species. J. Appl. Sci..

[53] Muthamilselvan T., Kuo T.-F., Wu Y.-C., Yang W.-C. (2016). Herbal remedies for coccidiosis control: A review of plants, compounds, and anticoccidial actions. Evid. Based Complement. Altern. Med..

[54] Asad M., Getachew A., Ahmad M. (2004). Antidiarrheal activity of methanolic extract of Rumex nervosus. J. Pharm. Res..

[55] Geetha M., Palanivel K. (2018). A Review on Poultry Coccidiosis. Int. J. Curr. Microbiol. App. Sci..

[56] Abudabos A.M., Alyemni A.H., Swilam E.O., Al-Ghadi M. (2017). Comparative Anticoccidial Effect of some Natural Products against *Eimeria* spp. Infection on Performance Traits, Intestinal Lesion and Occyte Number in Broiler. Pak. J. Zool..

[57] Tanweer A.J., Saddique U., Bailey C., Khan R. (2014). Antiparasitic effect of wild rue (*Peganum harmala* L.) against experimentally induced coccidiosis in broiler chicks. Parasitol. Res..

[58] Novaes J., Rangel L.T.L., Ferro M., Abe R.Y., Manha A.P., de Mello J.C., Varuzza L., Durham A.M., Madeira A.M.B., Gruber A. (2012). A comparative transcriptome analysis reveals expression profiles conserved across three *Eimeria* spp. of domestic fowl and associated with multiple developmental stages. Int. J. Parasitol..

[59] Abdisa T., Hasen R., Tagesu T., Regea G., Tadese G. (2019). Poultry Coccidiosis and its Prevention. Control. J. Vet. Anim. Res..

[60] Mitsch P., Zitterl-Eglseer K., Köhler B., Gabler C., Losa R., Zimpernik I. (2004). The effect of two different blends of essential oil components on the proliferation of Clostridium perfringens in the intestines of broiler chickens. Poult. Sci..

[61] Akanbi O.B., Taiwo V.O. (2020). The effect of a Local isolate and Houghton strain of Eimeria tenella on clinical and growth parameters following challenge in chickens vaccinated with IMMUCOX^®^ and LIVACOX^®^ vaccines. J. Parasit. Dis..

[62] Adarsh A., Chettiyar B., Kanthesh B., Raghu N. (2020). Phytochemical Screening and Antimicrobial Activity of “Cinnamon zeylanicum”. Int. J. Pharm. Res. Innov..

[63] El-Hack M.E.A., Alagawany M., Abdel-Moneim A.-M.E., Mohammed N.G., Khafaga A.F., Bin-Jumah M., Othman S.I., Allam A.A., Elnesr S.S. (2020). Cinnamon (*Cinnamomum zeylanicum*) Oil as a Potential Alternative to Antibiotics in Poultry. Antibiotics.

[64] Abbas R., Iqbal Z., Mansoor M. (2013). Role of natural antioxidants for the control of coccidiosis in poultry. Pak. Vet. J..

[65] Thangavel G., Mukkalil R., Chirakkal H. (2020). Plant Parts and Extracts Having Anticoccidial Activity. U.S. Patent.

[66] Abudabos A.M., Alyemni A.H., Hussein E.O., Al-Ghadi M.A.Q. (2018). Anticoccidial effect of some natural products in experimentally induced *Eimeria* spp. infection on carcass quality, intestinal lesion and ileal histology in broilers. J. Anim. Plant Sci..

[67] Adhikari P., Kiess A., Adhikari R., Jha R. (2020). An approach to alternative strategies to control avian coccidiosis and necrotic enteritis. J. Appl. Poult. Res..

